# Mr. Spurgeon on Hospitals

**Published:** 1887-06-25

**Authors:** 


### Mr. Spurgeon on Hospitals.

In seconding the first resolution passed at the Newington meeting in aid of the Hospital Sunday Fund, Mr. Spurgeon said :—I am delighted to see that you are getting your hands into action ready for next Sunday. You have taken them out of your pockets now, but put them in again next Sunday morning. I think that this Hospital Sunday Fund is acting very wisely in bringing the matter before the people. Truth, we know, is strong and will prevail; what we want is somebody to give a tongue to truth. There is many a great principle which has been lying by for hundreds of years, until somebody brought that principle out and put it into words, and kept on with it, worried, and it might be vexed, people, until at last that principle came to the front. It is no use having a Hospital Sunday Fund, and sitting down and expecting all the people in London to be looking out of their windows, eager to participate in assisting the Fund. You must put it under their noses, and make them see it. I hope the managers of this Fund will continue keeping this matter before the public, until everyone knows all about it. Then again, printers' ink is the very soul of business in helping on this kind of work. I find that the people need to be constantly told, and we must keep dinging away at them. It is no use trying to advocate a cause which everyone is ready to advocate. If anyone would only get up and say a hospital is a bad thing, and that the collection is a bad thing, then one could rise in eager defence, and show that one of the best things which humanity ever did for humanity, was to provide hospitals for the sick poor. Although, therefore, there is nothing to be said, I should like to say as much of it as possible. The needs of the hospitals will most assuredly grow. You have heard what Sir Sydney Waterlow said about the increased expenditure arising from more nurses being employed—better nursing—larger supplies of clean linen, and all sorts of things. But while these expenses grow, and must continue to grow, the incomes are getting smaller. I happen to be a Governor of St. Thomas's Hospital. Now, I saw on the agenda paper of the next meeting, that two or three items concerned the reduction of rents of certain farmers, who could not live if they paid the rents they formerly paid to the hospital. If I attend that meeting, I shall most certainly vote for the reduction; but then, you must remember, it is so much taken from the income of the hospital—another bed must be left unused. This is the case of one of our great hospitals which had to live on landed property, and the case is the same with others. I deplore the fact that their incomes are getting smaller by degrees and beautifully less. Therefore, we must arouse ourselves to make up this deficiency.

Observe how the population is growing. Why, in this south corner of London the children literally swarm in the streets. Where there used to be a nice quiet lane, now I find some "beautifully ugly" terrace which some builder, by the name of Jeremiah, has constructed, that we might be miserable. We must have more hospitals. I do not know whether we shall not be obliged to make the Government spend something in this direction. I don't believe in the Government doing anything well. I generally feel sorry when anything has to be left to the Government. I don't mean this Government in particular, but any Government which may be in office for the time being. It is six of one and half-a-dozen of the other. I have a very small opinion of the whole lot. There are some things which we should try ourselves to do as long as ever we can ; but if we are driven up in a corner, it may come to what I fear. Bones must be set, and the sick must be cared for ; the poor must not be left to die, in order not to have to go to the Government for help. So let us all try to give what we can. It is your duty to give, not merely as Christians, but as men. I like the Hospital Sunday movement, for all Christian people can meet, as we are met here to-night, on one platform. I believe I am a terrible "tiger" myself ; and as for Guinness Rogers, he is a lion that goes about seeking whom he can devour ; and my friend Mr. Palmer is a lamb ; but we shall not eat you up, for on such a point as this which we have met to urge we are all perfectly agreed.

There is a great deal to be done, and it is for all of us to see what we can do. I would to God that there was more everywhere of common kindness to those who are in need. The poor, I find, are as a rule very kind to one another, and I think sometimes a master might be better to the working man. There are times of pinching when he might send some little luxury and win that working man's heart, if he only wanted to see what he could do. We always feel kindnesses rendered to us and to our wives and children in times of need; therefore let us all aim to do what kindness we can for a poor man when he has to go to a hospital. To help his children when they are ill, surely cannot be an injury to anybody. You may talk about pauperising in giving them free relief : that is all very well ; but when I feel pain, when my tooth aches or my head aches, I do not care very much if you do pauperise me a little. I shall get my self-respect back when you ease me of that pain, when my tooth leaves off troubling me, or my head ceases to ache. Some people exhort others to give to the hospitals because they are helping themselves. Now I don't believe much in doing a thing merely to help yourself. I knew a family in Newcastle who, after being converted—so they said—joined the Church, and said they felt so grateful to God that they subscribed a sum of money to buy a teapot in commemoration of their conversion. Yes, a silver teapot for their own use in gratitude to God ! If you come next Sunday and give in that way,—well, we will accept your money, but at the same time I don't think there will be much about it that will be highly creditable to you, or acceptable to God. No; do it, my friends, because He has bidden you to love your neighbour as yourself. We do not, I fancy, recognise how near akin we are one to another. If we could look upon men and feel that God had made of one blood all nations of men, we should put our hands into our pockets to see what we could do for our poor brother Dick. May God bless every one of us to-night, and especially give us the blessing of a generous heart.

